# Polymorphisms of PPARα and ACTN3 Among Adolescent Egyptian Athletes: A Case–Control Study

**DOI:** 10.3390/life15030477

**Published:** 2025-03-16

**Authors:** Wael Ramadan, Rehan Monir, Ola El-Emam, Mohamed Diab, Dalia Shaheen

**Affiliations:** 1 Department of Sports Training, Faculty of Physical Education, Mansoura University, Mansura 35516, Egypt; mohamed-diab@mans.edu.eg; 2 Department of Medical Biochemistry, Faculty of Medicine, King Khalid University, Abha 62521, Saudi Arabia; rsoliman@kku.edu.sa; 3 Department of Medical Biochemistry, Faculty of Medicine, Mansoura University, Mansura 35516, Egypt; daliashaheen72@yahoo.com; 4 Department of Clinical Pathology, Faculty of Medicine, Mansoura University, Mansura 35516, Egypt; olaelemam@mans.edu.eg

**Keywords:** adolescent athletes, gene polymorphism, PPARA, ACTN3

## Abstract

Background: Athletic performance is a complex phenotype affected by individual traits, environmental conditions, training, and genetics. The peroxisome proliferator-activated receptor-alpha (PPARα) and alpha-actinin-3 (ACTN3) are two genes with the potential to influence human performance. The objective of the present study was to assess the genotype frequencies of ACTN3 (R/X) and PPARα (G/C) and to conduct a comparison of these frequencies among Egyptian adolescent athletes. Methods: This case–control study involved 228 individuals (118 elite-level athletes and 110 sedentary controls). Results: This study identified a statistically significant increase in the frequencies of the ACTN3 ‘R’ allele (77.5% compared to 55.9%; *p* < 0.001) and the PPARα ‘C’ allele (86.4% compared to 14.1%; *p* < 0.001) among athletes relative to the control groups. A similar pattern was noted for adolescent athletes in comparison to the control group in terms of both the R/R genotype (61.9% compared to 27.3%; *p* < 0.001) and the C/C genotype (80.5% compared to 2.7%; *p* < 0.001). In conclusion, these results imply that polymorphisms in ACTN3 and PPARα could be significant predictors for assessing the performance of adolescent Egyptian athletes.

## 1. Introduction

Athletic performance represents a multifaceted phenotype influenced by a range of genetic-, epigenetic-, environmental-, nutritional-, training-, equipment-, motivational-, and sleep-related factors [1]. Genetic variants can have a significant impact on the physiological aspects of athletic performance; however, determining the exact genetic underpinnings of such performance remains challenging [2]. These genetic polymorphisms, known as performance-enhancing polymorphisms (PEPs), may collectively impact the phenotypic characteristics of adolescent athletes [3].

Allelic association research is a case–control design utilized to identify genes contributing to physical performance. This research involves comparing the frequencies of alleles or genotypes of specific markers between adolescent athletes and a control group. When a positive connection is detected, the PEP may be investigated either by the actual functional variant or by close linkage to the functional allele [4,5]. Each contributing gene can only account for a small part of the variations observed between individuals. When several polymorphisms within one or more genes are investigated, the resultant findings are significantly more definitive [6,7,8].

The α-actinin-3 (ACTN3) and peroxisome proliferator-activated receptor-alpha (PPARα) genes illustrate complex interactions between genetic factors and performance outcomes. The DNA sequence for the human ACTN3 gene is situated on the long arm of chromosome 11, specifically at location 11q13-q14 [9].

The human ACTN3 gene codes for α-actinin-3, an actin-binding protein with a structural function in glycolytic type II muscle fibers. These fibers are responsible for generating force quickly and are involved in high-velocity movements. Additionally, α-actinin-3 plays a vital role in regulating muscle metabolism [10].

A common genetic single nucleotide polymorphism (SNP) located at codon 577 of the ACTN3 gene results in the substitution of an arginine (R) with a stop codon (X). The R allele denotes the conventional functional variant of the gene, whereas the X allele encompasses a sequence modification that entirely terminates the synthesis of the functional α-actinin-3 protein [11].

Consequently, this gene allele (XX) is associated with a poorer sprint/power performance, whereas the RR and RX alleles are linked to better sprint/power performance. The prevalence of the XX-null genotype seems to be higher in endurance athletes compared to controls [12,13], although contradictory findings have been reported [14,15].

The PPARα gene rs4253778 G/C (intron 7), located on chromosome 22, is a transcription factor responsible for regulating the equilibrium of glucose, lipids, and energy homeostasis [16]. It indirectly affects fatty acid metabolism by regulating gene expression and encoding many enzymes responsible for the oxidation of fatty acids, as lipid and carbohydrate metabolism are the primary sources of energy generation in skeletal muscle during movement. The expression level of PPARα is more significant in type I (slow-twitch) fibers than in type II (fast-twitch) muscle fibers [17,18].

The collective results of various studies have indicated that the combination of different forms of the PPARα and ACTN3 genes is associated with enhanced or diminished performance. This suggests that the variants of these genes may indeed be performance-enhancing polymorphisms (PEPs), which are believed to have a physiological impact on muscle fiber types, metabolism, and human body composition [19]. Moreover, previous studies on genetics in adolescent athletes have primarily concentrated on sports that fall at the extreme ends of the performance spectrum, namely sprint/power sports and endurance sports. However, there has been limited investigation into sports requiring a combination of aerobic and anaerobic energy systems, such as team sports or mixed-endurance sports [20]. Consequently, this research aims to test whether there are significant relationships among the genotypes of ACTN3 and PPARα for adolescent athletes and control groups. Additionally, the frequencies of alleles and genotypes among adolescent athletes versus control groups of the PPARα and ACTN3 polymorphisms have been studied.

## 2. Materials and Methods

**Study Design and Duration:** This research employs a case–control methodology, conducted over the period from January 2023 to February 2024.

**Target population**: The current study comprised 228 participants, categorized into two principal groups: Group A, consisting of adolescent athletes (N = 118) with an average age of 15.5 years (ranging from 13 to 18 years), and Group B, serving as the non-athletic control group (N = 110), with a mean age of 14.5 years (ranging from 13 to 16 years). All adolescent athletes had competed in national tournaments, whereas the control group included healthy adolescents with no history of competitive sports involvement. Eligibility criteria stipulated the absence of any diagnosed cardiorespiratory conditions among the participants. This study’s procedures conformed to the ethical principles delineated in the World Medical Association Declaration of Helsinki and adhered to ethical standards pertinent to research in exercise and sports science. Informed written consent was secured from all participants involved in our study, and for those individuals under the age of 18, consent was obtained from their legal guardians

**Study questionnaire**: An Arabic questionnaire was constructed to collect the following data during face-to-face interviews: age, sex, weight, and height. Venous blood was collected under aseptic conditions.

**Genotyping of ACTN3 and PPARα**

**Sample collection**

A blood sample of 2 mL was obtained from each participant via venipuncture and subsequently preserved in tubes containing ethylenediaminetetraacetic acid (EDTA). These samples were maintained at a temperature of −80 °C until they underwent further analysis.

**DNA Extraction**

The extraction of genomic DNA from the peripheral blood samples was carried out by utilizing the GeneJET Whole Blood Genomic DNA Purification Mini Kit (Thermo Scientific, Wilmington, DE, USA, Catalog Number K0781). The resultant DNA was subjected to quantification and purity assessment using a NanoDrop 2000 Spectrophotometer (Thermo Scientific, Wilmington, DE, USA).

**Genotyping**

Genotyping of the R577X polymorphisms in the ACTN3 gene, as well as polymorphisms in the PPARα gene, was conducted using polymerase chain reaction (PCR) complemented by restriction fragment length polymorphism (RFLP) analysis. PCR amplification was executed with a thermal cycler (The Applied Biosystems model 2720 thermal cycler is produced by Thermo Fisher Scientific, which is headquartered in Waltham, MA, USA).

**For the ACTN3 R577X gene**: Initial denaturation occurred for 5 min at 95 °C, followed by 35 cycles of denaturation for 30 s at 95 °C, annealing for 30 s at 60 °C, and extension for 30 s at 72 °C, concluding with a final extension for 10 min at 72 °C.

**For the PPARα gene**: The initial denaturation was performed for 8 min at 95 °C, followed by 35 cycles of denaturation for 1 min at 95 °C, annealing for 45 s at 50 °C, and extension for 45 s at 72 °C, culminating in a final extension for 10 min at 72 °C. The sequences of the primers employed (Invitrogen by Thermo Fisher Scientific, USA) and PCR product details are presented in Table 1. All PCR products were visualized on a 2% agarose gel stained with ethidium bromide.

**Table 1:** Primer sequence and PCR products of the studied SNPs.

	Primers’ Sequence	Annealing Temp.	Sources	PCR Product
ACTN3	F 5′-CTGTTGCCTGTGGTAAGTGGG-3′	60	Pereira et al. (2013) [21]	291 bp
	R 5′TGGTCACAGTATGCAGGAGGG-3′			
PPARα	F 5′-ACAATCACTCCTTAAATATGGTGG-3′	50	Paoli et al. (2014) [22]	266 bp
	R 5′-AAGTAGGGACAGACAGGACCAGTA-3′			

The restriction analysis of PCR products was performed utilizing the fast digest restriction enzyme Dded I (FastDigest HpyF3I (Dded I) by Thermo Scientific, USA) and TaqI (FastDigest TaqI by Thermo Scientific, USA) for ACTN3 and PPARα respectively as in Table 2.

**Table 2:** Restriction enzymes and restriction products of the studied SNPs.

SNP	Restriction Enzyme	Temperature	Restriction Product
ACTN3 R577X gene	FastDigest HpyF3I (Dded I)	37 °C	RR 205 and 86 bp, XX 108, 97, and 86 bp. RX 205 and 108, 97, and 86 bp.
PPARα rs4253778	FastDigest TaqI	65 °C	GG 266 CC 216, 50 GC 266, 216, and 50 bp

**Statistical Analysis:**

The collected data were examined using SPSS software (Version 25.0). To assess whether the population was representative, the Hardy–Weinberg equilibrium (HWE) was evaluated. Chi-square was employed to compare allele and genotype frequencies between the case–control cohorts. A *p*-value of ≤0.05 was deemed to indicate statistical significance.

## 3. Results

In this case–control study, a total of 228 participants were analyzed to assess the allelic and genotypic frequencies of the ACTN3 R577X and PPARα polymorphisms between adolescent athletes and a control group. The cohort comprised 118 adolescent athletes and 110 individuals in the control. Detailed demographic characteristics of the participants are presented in Table 3. Notably, the analysis revealed a statistically significant difference in age between the adolescent athletes and the control group. The two groups were also examined for Hardy–Weinberg equilibrium (HWE), as detailed in Table 4.

**Table 3:** Age, sex, and BMI in the two groups.

Characteristic	Athlete Group N = 118	Control Group N = 110	*p*
Sex			
Male	77 (65.3%)	68 (61.8%)	#0.590
Female	41 (34.7%)	42 (38.2%)	
BMI			
<25 kg/m^2^	90 (76.3%)	73 (66.4%)	#0.098
≥25 kg/m^2^	28 (23.7%)	37 (33.6%)	
Median	21.9	23.4	$0.022
(Q1–Q3)	(20.6–24.9)	(21.5–25.8)	
Age (years)	15.5 (13–18)	14.5 (13–16)	$ < 0.001

Categorical data are N (%), and test of significance is #chi-square test. Quantitative data are median (Q1–Q3), and test of significance is $Mann–Whitney U-test. *p* represents significance between athlete group and control group.

**Comparison of ACTN3 SNP between Two Groups**

A statistically significant elevation in the R/R genotype among adolescent athletes compared to control groups was observed (P1 < 0.001), as shown in Table 5.

A statistically significant increase in the frequency of the ACTN3 ‘R’ allele was detected in athletes compared to the control group (*p* < 0.001). Individuals possessing the ACTN3 ‘R’ allele exhibit 2.7 times greater likelihood of being adolescent athletes, as indicated in Table 6.

The inheritance model demonstrating the lowest *p*-value, AIC, and BIC was chosen as the optimal model. The most suitable inheritance model for ACTN3 was identified as the recessive model (R/R vs. X/X-X/R). Participants with the R/R genotype have 6.7 times higher odds of being part of the athlete group relative to those with either the X/X or X/R genotype, after adjusting for age, sex, and BMI, as presented in Table 4.

**Comparison of PPARα SNP between Two Groups**

Statistical analyses substantially elevated the C/C genotype compared to the C/G and G/G genotypes in adolescent athletes versus control groups. Additionally, we observed statistically significant differences between the two groups, as depicted in Table 5.

There was a statistically significant presence of the PPARα ‘C’ allele in athletes compared to the control groups. Individuals with the PPARα ‘G’ alleles have 38.9 times higher odds of being athletes as indicated in Table 6. Participants with the C/C genotype have 2943 times higher odds of being athletes compared to those with either the G/G or G/C genotype, adjusted for age, sex, and BMI, as shown in Table 4.

**Table 4:** ACTN3 and PPARα Hardy–Weinberg equilibrium (HWE) and best inheritance model.

Gene	Best Model	Genotypes	Athlete Group	Control Group	AORs (95% CI)	*p*-Value	AIC	BIC
ACTN3	Recessive	X/X-X/R R/R	45 (38.1%) 73 (61.9%)	80 (72.7%) 30 (27.3%)	r(1) 6.7 (3–15.1)	<0.001	282.6	594.7
PPARα	Recessive	G/G-G/C C/C	23 (19.5%) 95 (80.5%)	107 (97.3%) 3 (2.7%)	r(1) 2943 (229.4–37761)	<0.001	221.1	533.2

Notes: AORs = adjusted odds ratio (for age, sex, and BMI), CI = confidence interval, AIC = Akaiki information criterion, BIC = Bayesian information criterion, r(1) = reference category.

**Table 5:** ACTN3 and PPARα genotypes in the two groups.

Characteristic	Athlete Group N = 118	Control Group N = 110	*p*
ACTN3			
R/R	73 (61.9%)	30 (27.3%)	<0.001
R/X-X/X	45 (38.1%)	80 (72.7%)	
z-tests	a	a	
PPARα			
C/C	95 (80.5%)	3 (2.7%)	<0.001
C/G-G/G	23 (19.5%)	107 (97.3%)	
z-tests	a	a	

Notes: Data are N (%), and test of significance is chi-square test.

**Table 6:** Allele frequencies in the two groups.

Gene	Athlete Group	Control Group	Chi-Square Test of Association	Binary Logistic Regression			
	N = 118	N = 110	χ2	ϕ	*p*-Value	COR (95% CI)	*p*-Value
ACTN3			24.14	0.23	<0.001		<0.001
‘R’ allele	183 (77.5%)	123 (55.9%)				2.7 (1.8–4.1)	
‘X’ allele	53 (22.5%)	97 (44.1%)				r(1)	
PPARα			238.62	0.723	<0.001		<0.001
‘C’ allele	204 (86.4%)	31 (14.1%)				38.9 (22.8–66.2) r(1)	
‘G’ allele	32 (13.6%)	189 (85.9%)					

Data are N (%), (χ2) chi-square test of association; (ϕ) binary logistic regression, COR = crude odds ratio, CI = confidence interval, r(1) = reference category.

## 4. Discussion

Athletic performance constitutes a multifaceted phenotype influenced by a synthesis of individual, environmental, nutritional, training, and genetic determinants. Two genes that may affect human performance, muscle function, and metabolism are PPARα and ACTN3 [2]. The examination of genotype distributions and allele frequencies of the ACTN3 gene revealed a statistically significantly higher ACTN3 ‘R’ allele frequency (77.5% vs. 55.9%; >0.001) in athletes compared to the control group. Moreover, our analysis revealed a statistically higher prevalence of the R/R athlete group compared to the control group. These findings are aligned with prior research. Yang et al. [10] established a significant association between the ACTN3 genotype and athletic performance, demonstrating that elite sprinters exhibited significantly elevated frequencies of the 577R allele relative to the control group [10]. Similarly, Santiago et al. [23] conducted a study in Spain that revealed that the proportions of the 577RR and 577RX genotypes (48.3% and 36.7%, respectively) were notably different from those in the control group (n = 123; 28.5% and 53.7%) and endurance athletes (n = 52; 26.5% and 52%), with this difference reaching statistical significance (*p* = 0.041) [23]. Meanwhile, Pimenta et al. [24] investigated a group of 200 professional soccer players from Brazil. They discovered that individuals who carried the 577RR genotype could run faster than those with the 577XX genotype, specifically at distances of 10, 20, and 30 m. Subsequent research has demonstrated a strong association between adolescent power performance and the RR genotype [24,25].

Furthermore, the ACTN3 RR genotype may enhance power performance and speed, whereas the ACTN3 XX genotype might contribute to endurance performance [26]. Eynon et al. [27] undertook a comprehensive analysis to compare the frequencies of the ACTN3 R577X polymorphisms across a substantial cohort comprising team sport athletes (n = 205), endurance athletes (n = 305), sprint/power athletes (n = 378), and non-athletic controls (n = 568) from Europe. The findings of this study indicated no significant association between the ACTN3 R577X polymorphism and athletic status related to team sports [27]. However, it was notably documented that the 577RR genotype had a higher prevalence among sprint/power athletes compared to team sport athletes. Another meta-analysis corroborated the idea that athletes with higher speed and power exhibited a higher occurrence of the RR genotypes [12].

Our findings align with those of Kikuchi et al. [28], corroborating the link between the RR + RX genotype of the ACTN3 R577X polymorphism and the status of elite sprint/power athletes, as well as the association of the ACTN3 RR + RX genotype with proficiency in long-distance running [28]. Furthermore, Akazawa et al. [29] observed that the frequency of the R allele genotype is more prevalent among sprint/power athletes compared to those in endurance sports and control groups.

Additionally, Yang et al. [10] identified substantial differences in ACTN3 alleles and genotypes between elite and sub-elite athletes occupying various positions, with a notable association between the RR genotype and power-related athletic performance among young Chinese football players [30]. These findings imply that the presence of R alleles in the ACTN3 R577X polymorphism correlates with a predisposition toward sports that demand enhanced muscular strength. The RR genotype may significantly contribute to attaining international-level proficiency in sports reliant on anaerobic metabolism, such as sprinting and power disciplines.

The role played by the ACTN3 genotype in influencing skeletal muscle performance among elite athletes and in facilitating adaptation to varying physical demands in the general population can be ascribed to the critical function of ACTN3 in regulating the synthesis and degradation of muscle proteins, in addition to ensuring the optimal maintenance of muscle mass homeostasis from early developmental stages [31].

In contrast, the research conducted by Garatachea et al. [32] did not demonstrate any correlation between ACTN3 genotypes and explosive leg power among elite basketball players, nor did it establish a connection between these genotypes and the athletes’ classification within this sports category. Additionally, no significant differences in genotype frequencies were observed between control subjects and endurance athletes [32], as supported by other studies [33]. This inconsistency may be attributed to variations in sample size, ethnic diversity, and lifestyle differences.

Conversely, Tural et al. [34] reported a statistically significant distinction in a Turkish study between the endurance athletes and control groups regarding the distribution of the PPARα ’GG’ genotype (*p* = 0.006) and G allele frequencies (*p* = 0.001) [34]. Furthermore, elite endurance athletes tend to exhibit a higher prevalence of the GG genotype and G allele [35].

Humińska-Lisowska et al. [36] demonstrated that the prevalence of the PPARα GG genotype (73.33% versus 54.70%, *p* = 0.04) and the G allele (82.50% versus 70.17%, *p* = 0.01) was significantly higher among elite combat athletes than in control groups. Furthermore, research by Maciejewska-Skrendo et al. [8] indicated that the GG genotype is more common in endurance athletes, suggesting that the G allele may be one of the alleles linked to endurance capability. Additionally, Petr et al. [37] identified an association between the C allele in PPARα rs4253778 and participation in soccer, while the G allele in PPARα rs4253778 was correlated with elite endurance athlete status.

This discrepancy can be attributed to the presence of a C/G polymorphism in intron 7, a non-coding region of the PPARα gene. It is highly probable that this polymorphism is not operational. However, it might be associated with a functional variant in the promoter of the enhancer element of the PPARα gene, leading to its expression. Moreover, research has demonstrated that this gene interacts with other variations in the peroxisome proliferator-activated receptors [38].

When examining the potential role of genetics in adolescent athletes, it is crucial to consider the physiological demands placed on an athlete’s body, which necessitate managing energy sources and maintaining equilibrium between glucose metabolism and fatty acid utilization, particularly regarding metabolic stress during sustained or short-term and intensive activities [39,40]. PPARα and ACTN3 function as molecular sensors that regulate fatty acid metabolism and transport across various tissues, potentially impacting the allocation of energy substrates. According to biochemical investigations, under physiological conditions, PPARα agonists enhance the transfer of fatty acids from the liver and skeletal muscle to adipose tissue. These mechanisms lead to decreased glucose production in the liver, increased glucose utilization in the muscles via the Randle cycle, and reduced fatty acid metabolism in the aforementioned tissues [41]. Insulin-dependent signaling pathways, functioning within a complex network, regulate most of these effects. Elevated glucose utilization in active skeletal muscles may be one of the most crucial factors for athletes engaged in short-term activities. The anaerobic system is recognized as vital for energy generation in sports that require throwing, jumping, lifting, and short sprints. In anaerobic metabolism, glucose is the most significant resource, as it is essential for glycolysis to provide the requisite amount of energy for very rapid (20–30 s) and intense efforts [42].

The results of our study demonstrate that athletes displayed a markedly greater prevalence of the RR/CC genotype and the R/C allele. This inference is derived from our examination of genotype and allele distribution and frequency among the athletic cohort in comparison to the control group. It is crucial to acknowledge, however, that various factors, such as environmental influences, gene interactions, and gene–environment interplay, substantially affect athletic performance. These factors also contribute significantly to the “complex trait” of achieving adolescent athletic status and cannot be solely attributed to the identification of genetic polymorphisms.

**Limitations of the current study**: An in-depth analysis of the association between PPARα and ACTN3 polymorphisms and physical performance should be repeated in further large-scale future studies.

## 5. Conclusions

The findings of this study suggest that polymorphisms in the ACTN3 and PPARα genes may serve as important indicators for evaluating the athletic performance of adolescent Egyptian athletes. This study identified a statistically significant higher prevalence of the ACTN3 ’R’ allele (77.5% compared to 55.9%; *p* < 0.001) and the PPARα ’C’ allele (86.4% compared to 14.1%; *p* < 0.001) in athletes as opposed to control groups. A similar pattern was noted among adolescent athletes relative to the control group, particularly in the frequencies of the R/R genotype (61.9% compared to 27.3%; *p* < 0.001) and the C/C genotype (80.5% compared to 2.7%; *p* < 0.001). Consequently, while genetic testing may offer valuable insights into the association between genetic polymorphisms and physical performance potential, further research involving Olympic athletes from diverse populations is warranted.

## Data Availability

The original contributions presented in this study are included in the article. Further inquiries can be directed to the corresponding author.
